# Supplementary figures and images for: Spatiotemporal control of actomyosin contractility by MRCKβ signaling drives phagocytosis
Source: J Cell Biol. 2022 Sep 19;221(11):e202012042. doi: 10.1083/jcb.202012042 (PMC9485704; doi:10.1083/jcb.202012042)

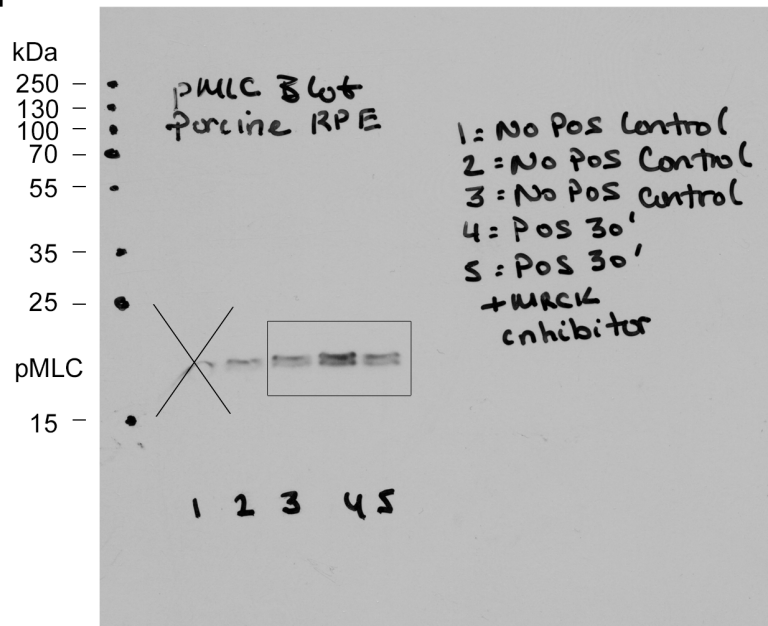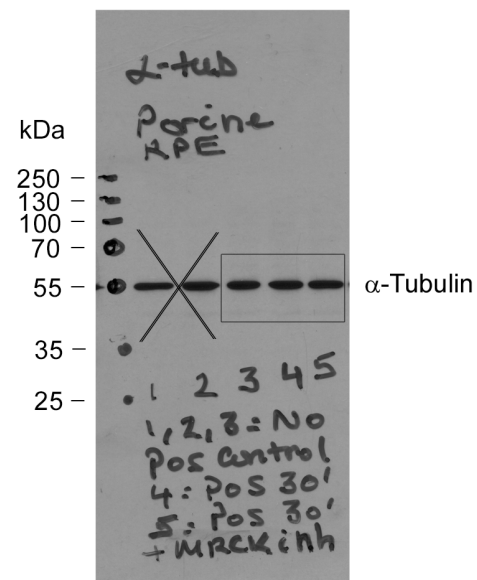

m

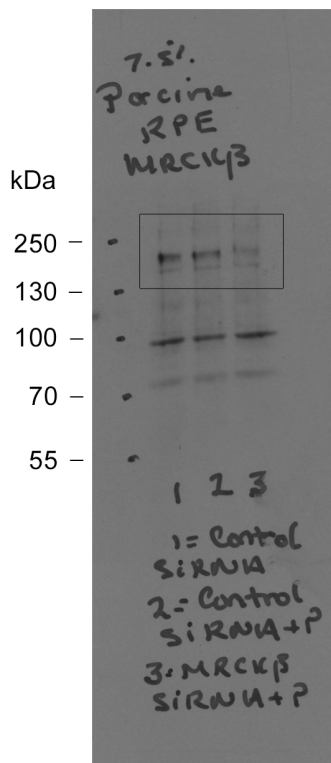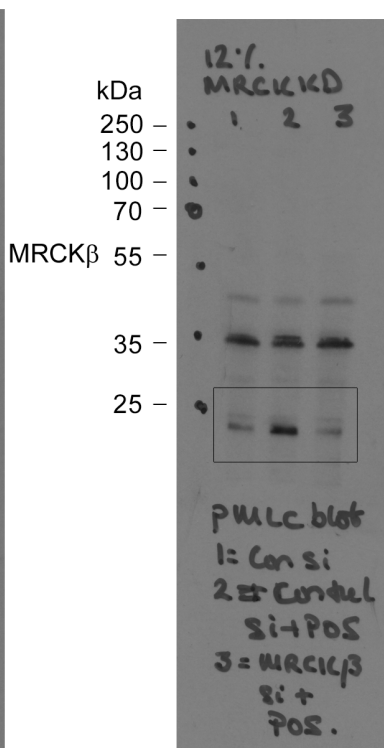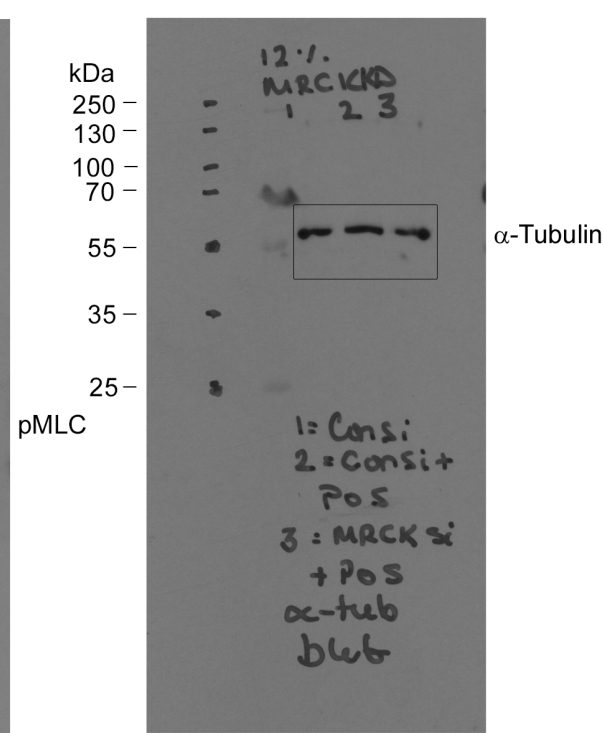

Supplement: SourceData F3 — contains original blots for Fig. 3. [file JCB_202012042_SourceDataF3.pdf]

p

kDa

250 -  
130 -  
100 -  
70 -  
55 -  
35 -  
25 -

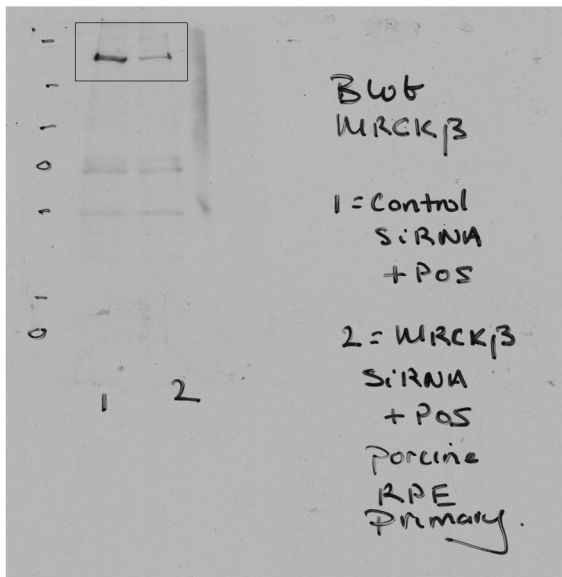

MRCK $\beta$

kDa

250 -  
130 -  
100 -  
70 -  
55 -  
35 -  
25 -

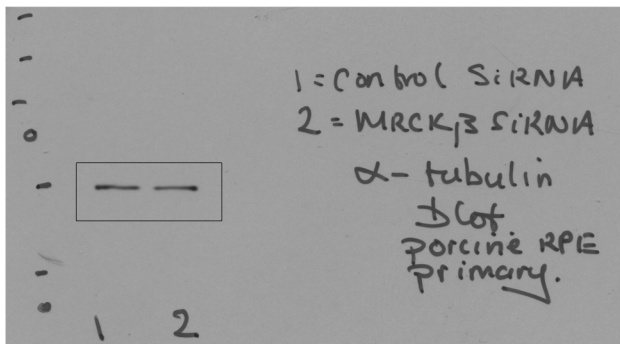

$\alpha$ -Tubulin

Supplement: SourceData F4 — contains original blots for Fig. 4. [file JCB_202012042_SourceDataF4.pdf]

n

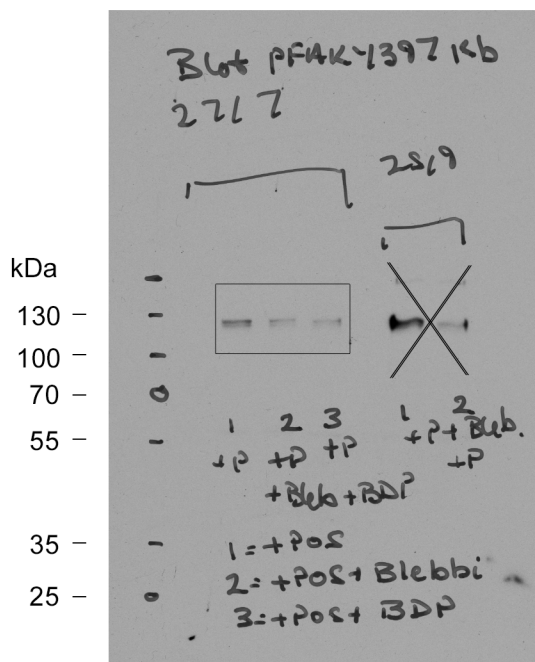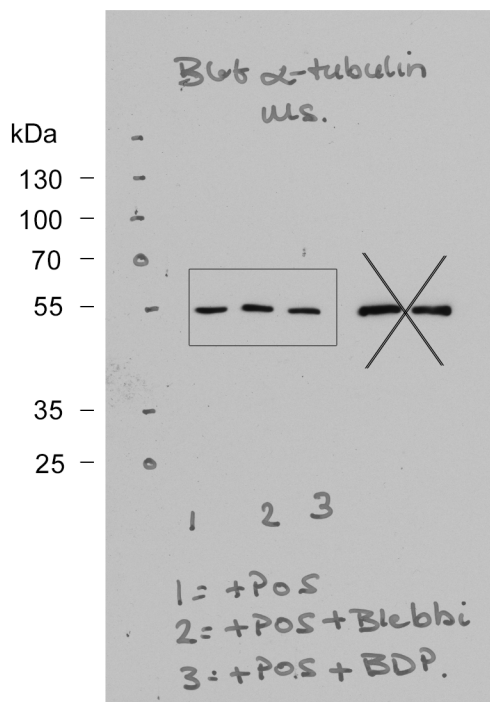

o

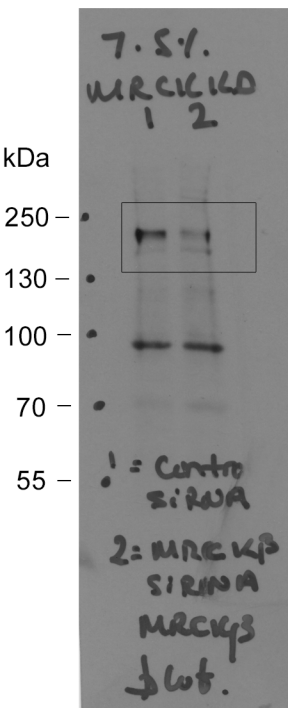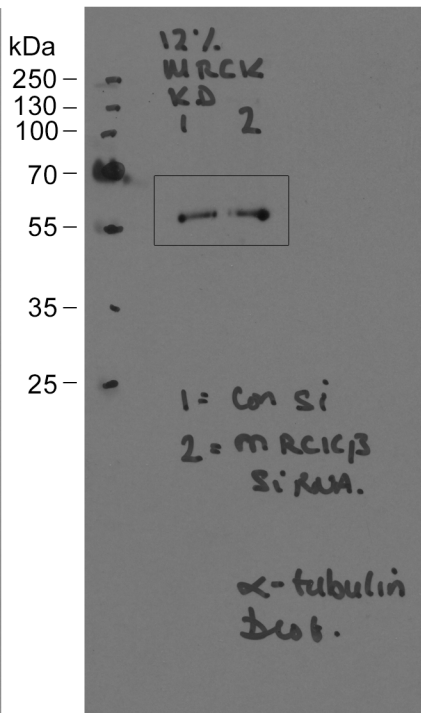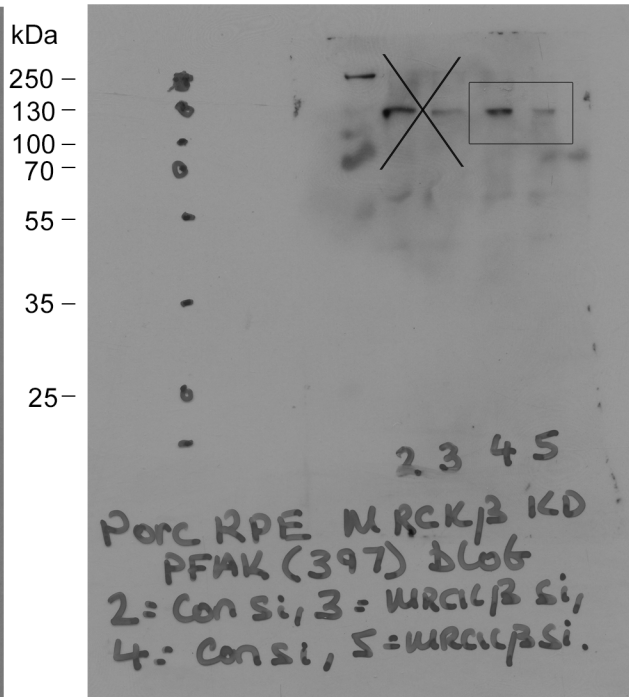

Supplement: SourceData F6 — contains original blots for Fig. 6. [file JCB_202012042_SourceDataF6.pdf]

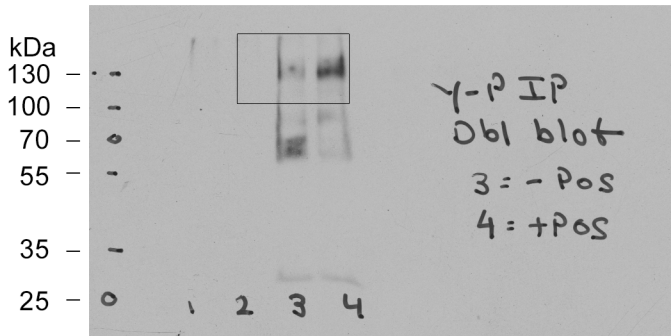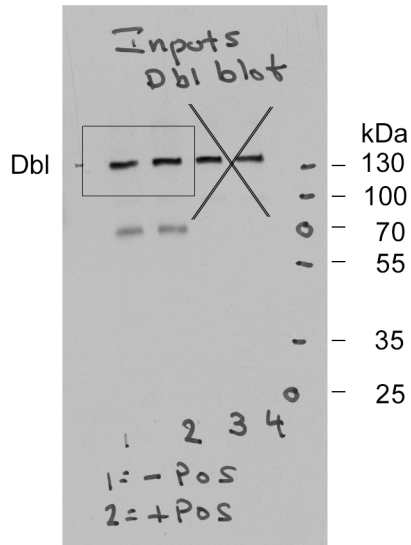

Supplement: SourceData F8 — contains original blots for Fig. 8. [file JCB_202012042_SourceDataF8.pdf]

c

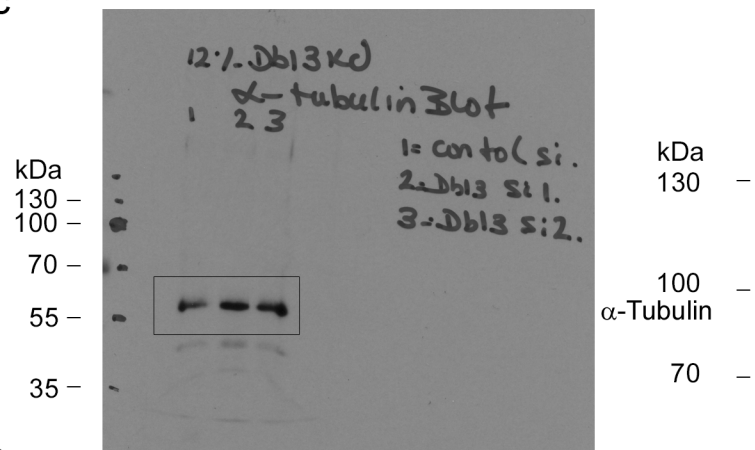

e

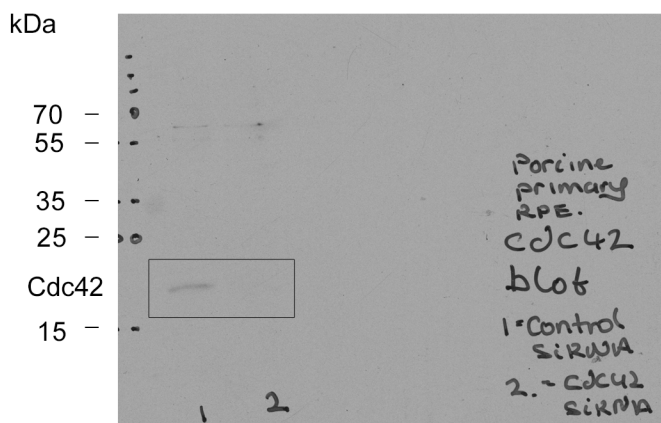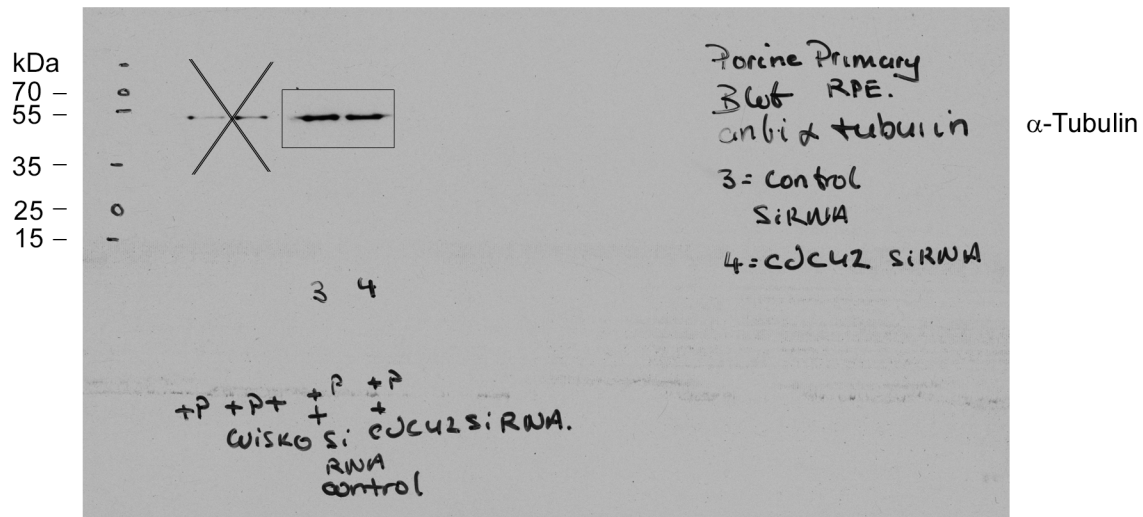

k

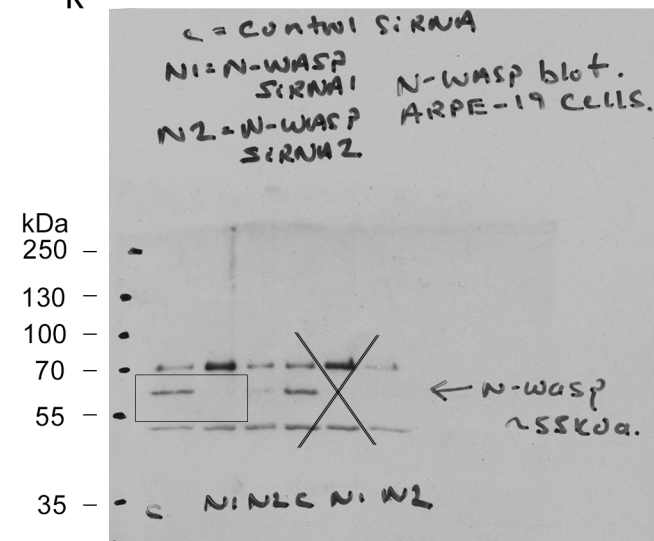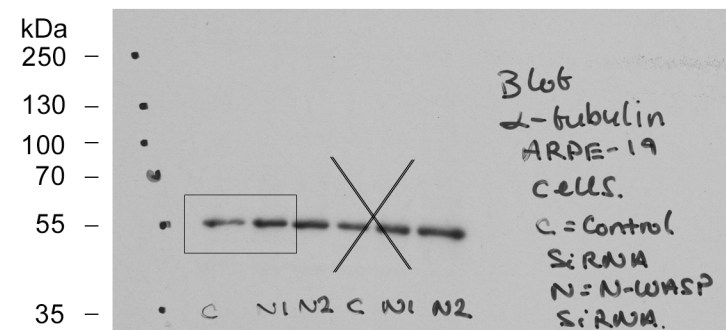

Supplement: SourceData FS1 — contains original blots for Fig. S1. [file JCB_202012042_SourceDataFS1.pdf]

C

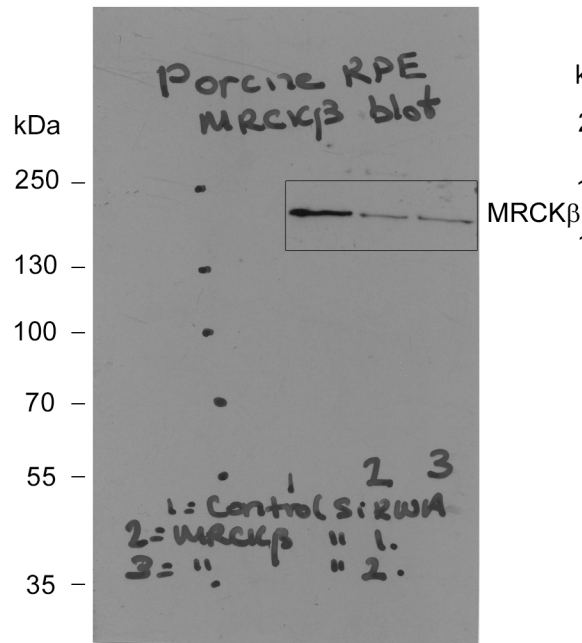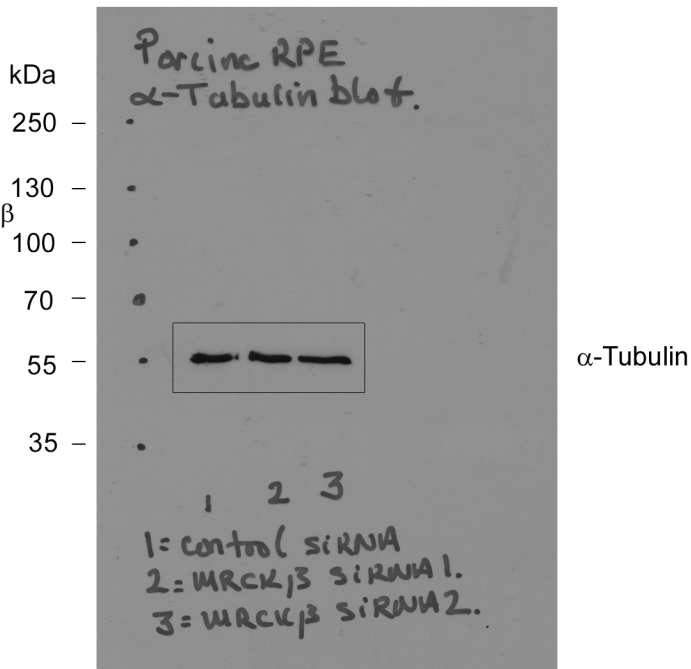

Supplement: SourceData FS3 — contains original blots for Fig. S3. [file JCB_202012042_SourceDataFS3.pdf]

1

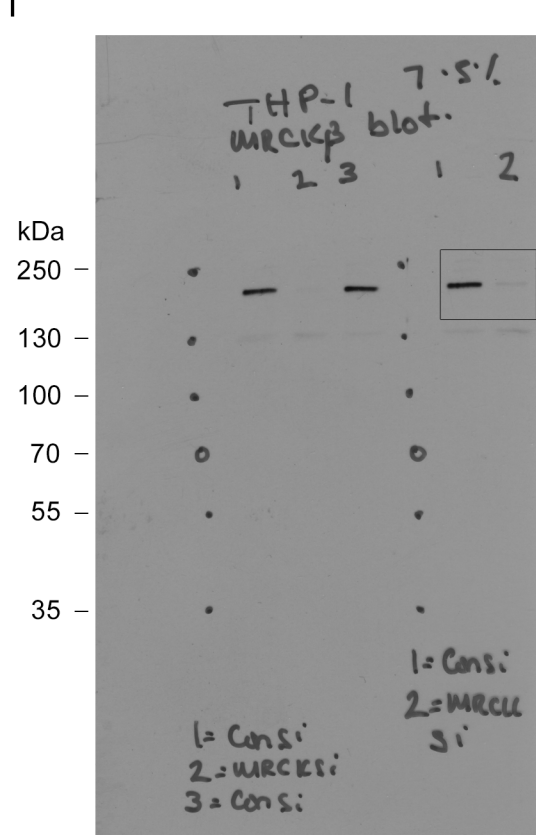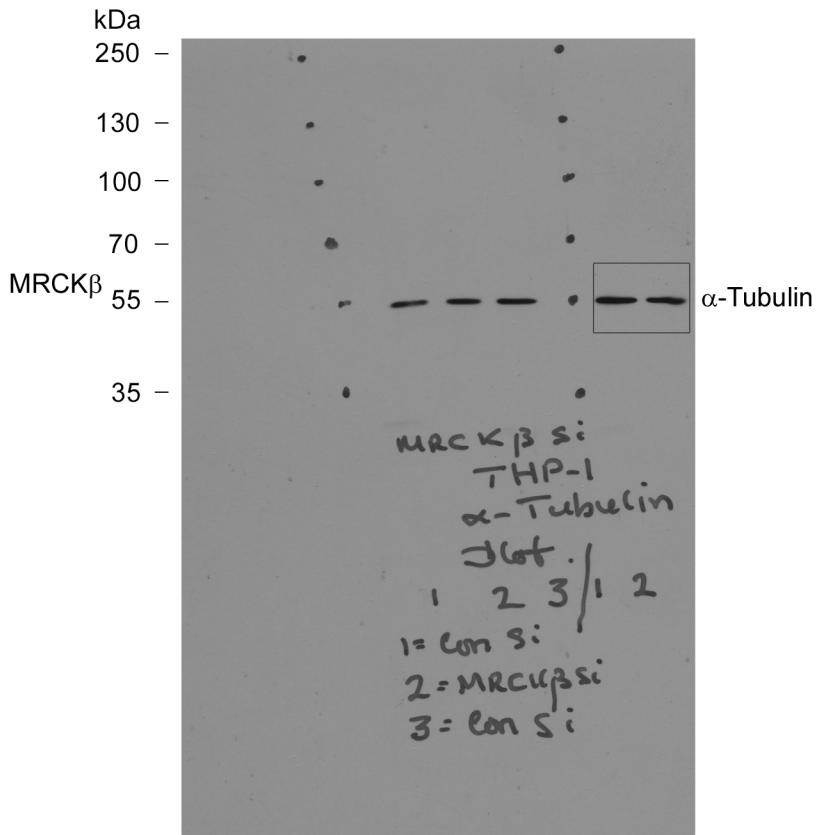

Supplement: SourceData FS5 — contains original blots for Fig. S5. [file JCB_202012042_SourceDataFS5.pdf]
